# British Hospitals Association

**Published:** 1912-10-12

**Authors:** 


### British Hospitals Association.

The Newly Elected Council.

*Chairman of the Council.*

D. J. Mackintosh, M.B., LL.D., M.V.O., Superintendent, Western Infirmary, Glasgow.

*Members of the Council.*

E. C. Barnes, Chairman, Chesterfield and North Derbyshire Hospital.

J. C. Buchanan, Secretary and House Governor, Metropolitan Hospital, Kingsland, London.

Sir Henry Burdett, K.C.B., K.C.V.O., London.

Rev. J. Montgomery Campbell, Chairman, Royal Infirmary, Dumfries, N.B.

W. G. Carnt, Superintendent, Royal Infirmary, Manchester.

Albert Cay, Treasurer, Warneford Hospital, Leamington.

Howard J. Collins, House Governor, General Hospital, Birmingham.

Rev. G. B. Cronshaw, Chairman, Radcliffe Infirmary, Oxford.

H. Wade Deacon, Secretary, Liverpool Council of Voluntary Aid.

Lieut.-Colonel Sir Joseph Fayrer, Bart., F.R.C.S., M.D., Superintendent, Royal Infirmary, Edinburgh.

Edmund Forster, M.A., Superintendent, Royal Infirmary, Derby.

G. E. Friend, Superintendent, St. George's Hospital, London.

Thomas Hayes, Clerk, St. Bartholomew's Hospital, London.

J. D. Hedderwick, Chairman, Royal Infirmary, Glasgow.

Evelyn Shaw Hellier, Chairman, General Hospital, Wolverhampton.

R. J. Johnstone, F.R.C.S., Royal Victoria Hospital, Belfast.

Charles Lupton, Treasurer, General Infirmary, Leeds.

Thomas Maddock, Hon. Secretary, Cottage Hospital, Wellingborough.

Colonel J. A. Roxburgh, Western Infirmary, Glasgow.

W. A. Tait, Royal Infirmary, Edinburgh.

A. William West, Treasurer, St. George's Hospital, London.

Charles Yeomans, Treasurer, Jessop Hospital for Women, Sheffield.

*Hon. Treasurer.*

Stewart Johnson, The Hospital for Sick Children, Great Ormond Street, London, W.C.

*Hon. Secretary.*

Conrad W. Thies, 14 Belsize Park Gardens, Hampstead, N.W.

